# Integration of inhibitory and excitatory effects of α7 nicotinic acetylcholine receptor activation in the prelimbic cortex regulates network activity and plasticity

**DOI:** 10.1016/j.neuropharm.2016.02.028

**Published:** 2016-06

**Authors:** Matthew Udakis, Victoria Louise Wright, Susan Wonnacott, Christopher Philip Bailey

**Affiliations:** a Department of Pharmacy & Pharmacology, University of Bath, Bath BA2 7AY, UK; b Department of Biology & Biochemistry, University of Bath, Bath BA2 7AY, UK

**Keywords:** Nicotinic receptors, Prefrontal cortex, Prelimbic cortex, Glutamate, GABA, LTP

## Abstract

Cognitive and attentional processes governed by the prefrontal cortex (PFC) are influenced by cholinergic innervation. Here we have explored the role of α7 nicotinic acetylcholine receptors (nAChRs) as mediators of cholinergic signalling in the dorsomedial (prelimbic) PFC, using mouse brain slice electrophysiology. Activation of α7 nAChRs located on glutamatergic terminals and cell soma of GABAergic interneurons increased excitation and inhibition, respectively, in layer V of the prelimbic cortex. These actions were distinguished by their differential dependence on local acetylcholine (ACh): potentiation of endogenous cholinergic signalling with the positive allosteric modulator, PNU-120596, enhanced spontaneous excitatory events, an effect that was further increased by inhibition of acetylcholinesterase. In contrast, α7 nicotinic modulation of inhibitory signalling required addition of exogenous agonist (PNU-282987) as well as PNU-120596, and was unaffected by acetylcholinesterase inhibition. Thus α7 nAChRs can bi-directionally regulate network activity in the prelimbic cortex, depending on the magnitude and localisation of cholinergic signalling. This bidirectional influence is manifest in dual effects of α7 nAChRs on theta-burst-induced long-term potentiation (LTP) in layer V of the prelimbic cortex. Antagonism of α7 nAChRs significantly decreased LTP implicating a contribution from endogenous ACh, consistent with the ability of local ACh to enhance glutamatergic signalling. Exogenous agonist plus potentiator also decreased LTP, indicative of the influence of this drug combination on inhibitory signalling. Thus α7 nAChRs make a complex contribution to network activity and synaptic plasticity in the prelimbic cortex.

## Highlights

• α7 nAChRs exist at glutamatergic nerve terminals in the prelimbic cortex.
• α7 nAChRs exist at GABAergic cell bodies in the prelimbic cortex.
• Tonic ACh preferentially activates α7 nAChRs at glutamatergic nerve terminals.
• α7 nAChRs exert bidirectional control of LTP in the prelimbic cortex.

## Graphical abstract

**Figure:**
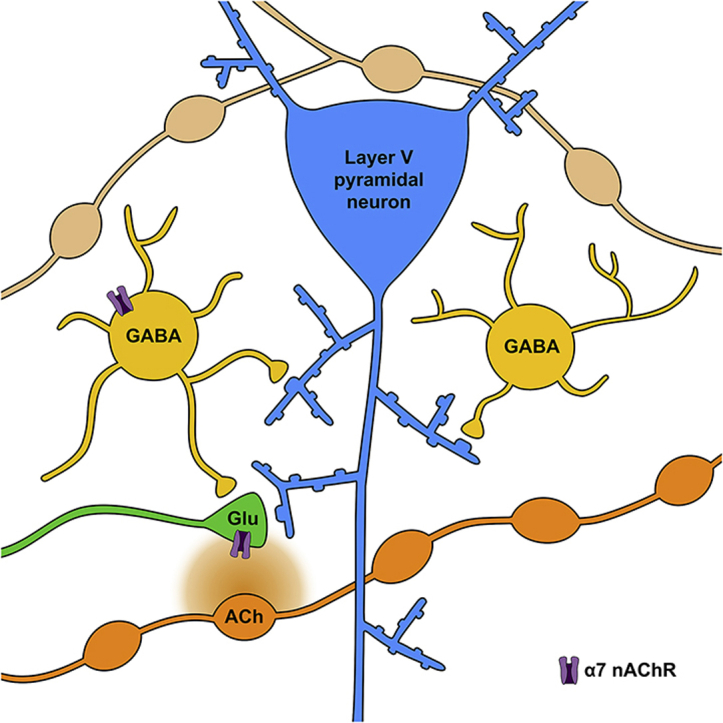

## 1 Introduction

The prefrontal cortex (PFC) is the locus for ‘executive’ function: cognitive processes that include working memory, attentional processing, decision making and behavioural inhibition (Briand et al., 2007). PFC dysfunction is implicated in many neuropsychiatric conditions, including schizophrenia, Alzheimer's disease, addiction and attentional disorders (Briand et al., 2007, Lewis, 2009, Van den Oever et al., 2010). The rodent medial PFC (mPFC) corresponds to the human dorsolateral PFC (Heidbreder and Groenewegen, 2003). Subregions of the mPFC have been ascribed differential roles, with the dorsomedial (prelimbic) area implicated in various social, emotional and goal-directed learning (Balleine and O'Doherty, 2010, Moorman et al., 2015). The mPFC receives glutamatergic inputs from many cortical and sub-cortical structures and integration of this information results in the generation of outputs from pyramidal neurons in layers V/VI. Glutamatergic synapses within the mPFC can exhibit activity-dependent changes in synaptic strength (synaptic plasticity), providing a cellular paradigm for cognitive processes (Laroche et al., 2000). Key to network integration within the PFC are local GABAergic interneurons and it is increasingly recognised that this network is modulated by ascending neurotransmitter systems (Briand et al., 2007).

Cholinergic projections from the basal forebrain innervate all layers of the mPFC (Bloem et al., 2014b) with ACh implicated in attentional and cognitive processes (Parikh and Sarter, 2008, Bloem et al., 2014a). The actions of ACh are mediated by multiple subtypes of muscarinic and nicotinic acetylcholine receptors (nAChRs); improvements in attention, working memory and executive processes elicited by nicotine and subtype-selective nicotinic agonists has shifted focus onto the actions of nAChRs (Wallace and Bertrand, 2013). The major nAChR subtypes, heteromeric α4β2 and homomeric α7 nAChRs, are expressed in the PFC and credited with pro-cognitive properties. The non-selective agonist nicotine alters the threshold of spike-timing dependent plasticity at glutamate synapses by enhancing mPFC inhibition, an effect attributed to activation of α4β2 and α7 nAChRs on inhibitory interneurons in layers II/III and V (Couey et al., 2007). α4β2 and α7 nAChRs are also present on pyramidal neurons in layers VI and V respectively (Bloem et al., 2014a) and on excitatory amino acid-releasing boutons in the PFC (Dickinson et al., 2008).

The actions of endogenous ACh, as opposed to exogenous agonist, are more challenging to study. However, type II positive allosteric modulators (PAMs) of α7 nAChRs, such as PNU-120596, allow the effects of endogenous ACh to be disclosed by preventing desensitization as well as enhancing agonist responses (Hurst et al., 2005, Livingstone et al., 2009). In this study we have applied PNU-120596, in the presence and absence of an α7-selective agonist, to explore the role of α7 nAChRs in modulating synaptic transmission in the mouse prelimbic cortex. We show that α7 nAChRs can enhance both glutamatergic and GABAergic signalling by distinct mechanisms, giving them a bidirectional role in prelimbic cortex neurotransmission. Endogenous ACh release selectively targets α7 nAChRs that enhance glutamate release, whereas global activation of α7 nAChRs by exogenous agonist was required to enhance inhibitory signals. Through this complex influence on neurotransmission α7 nAChRs can alter synaptic plasticity and network function within the prelimbic cortex.

## 2 Materials and methods

### 2.1 Slice preparation

Male C57BL/6 mice, 5 weeks old and bred at the University of Bath, were anaesthetised via intraperitoneal injection of 160 mg/kg ketamine and 20 mg/kg xylazine and then decapitated. All mice used were wild-type with the exception of recordings from GABAergic interneurons where heterozygote GAD67-GFP mice were used (Tamamaki et al., 2003). Brains were immediately removed and submerged in ice-cold cutting solution containing in mM: 20 NaCl, 2.5 KCl, 1.6 NaH_2_PO_4_, 7 MgCl_2_, 0.5 CaCl_2_, 60 NaHCO_3_, 24 d-glucose, 85 sucrose with an osmolarity of 300 mOsm/L and saturated with 95% O_2_/5% CO_2_. Coronal brain slices (285–400 μm thickness, 1.54–2.34 mm anterior to bregma) containing the mPFC were obtained using a vibratome (DSK, DTK-1000). Slices were then incubated at 32 °C for 30 min in artificial cerebrospinal fluid (aCSF) containing in mM: 125 NaCl, 2.5 KCl, 1.2 NaH_2_PO_4_, 1.2 MgCl_2_, 2.4 CaCl_2_, 21.4 NaHCO_3_, 11.1 d-glucose, 0.1 ascorbic acid, with an osmolarity of 300 mOsm/L and saturated with 95% O_2_/5% CO_2._ Slices were maintained at room temperature for at least a further 30 min before commencing the experiment. All protocols and procedures were in accordance with the UK Animals (Scientific Procedures) Act 1986, the European Communities Council Directive 1986 (86/609/EEC), the ARRIVE guidelines (Kilkenny et al., 2010) and the University of Bath ethical review document. All efforts were made to minimise animal suffering and to limit the number of animals used.

### 2.2 Whole cell patch-clamp experiments

Slices (285–300 μm) were transferred to a submerged chamber with a continuous flow rate of 2–3 ml/min aCSF saturated with 95% O_2_/5% CO_2_ at 32 °C. Slices were visualised using oblique optics on an Olympus BX51WI upright microscope. Prelimbic layer V pyramidal neurons were identified as being approximately 300–500 μm from the slice midline and possessing a pyramidal neuron morphology. Recording electrodes with a 2–5 MΩ resistance were fabricated using a micropipette puller (Sutter-instruments, P-97). For the Whole cell voltage clamp recordings the intracellular recording solution contained in mM: 120 Cs methanesulphonate, 10 NaCl, 2 MgCl_2_, 10 HEPES, 0.5 EGTA, 5 QX314, 2 Mg-ATP, 0.25 Na-GTP, with an osmolarity of 285 mOsm/L.

All recordings of spontaneous and miniature excitatory post-synaptic currents (EPSCs) and inhibitory post-synaptic currents (IPSCs) were performed in the absence of GABAergic or glutamatergic blockers, to ensure the continued presence of local network activities. To achieve this, holding voltages were alternated throughout the recordings from −60 mV to 0 mV (respectively the reversal potentials of GABA_A_ and AMPA receptor-mediated currents) (Semyanov and Kullmann, 2000). At Vh = −60 mV, all spontaneous events were blocked by DNQX (50 μM), and at Vh = 0 mV, all spontaneous events were blocked by picrotoxin (50 μM) (data not shown). Recordings were amplified and filtered at 2 kHz (Axopatch 200A amplifier, Axon Instruments), and digitised with a sampling rate of 10 kHz (Digidata 1440 A, Axon Instruments). For spontaneous and miniature EPSC and IPSC experiments, drugs were applied via perfusion to the slice for at least 5 min before event measurement. Miniature EPSCs and IPSCs (mEPSC, mIPSC) were recorded in the presence of tetrodotoxin (TTX, 1 μM). Stimulated EPSCs were evoked via a stimulus pulse (0.05 Hz, 0.15 ms) using a metal bipolar stimulating electrode. Stimulus intensities that gave 25–50% maximum response were used for the entire recording. Responses were then averaged to give an average response per minute. For all patch clamp experiments, series resistance was measured throughout experiments and data were excluded if series resistance changed by 25%. A liquid junction potential of ∼12 mV was calculated using pCLAMP software (Axon instruments) and accounted for during the experiments.

For whole cell current clamp recordings of interneurons the intracellular solution contained in mM: 120 K-gluconate, 20 KCl, 2 MgCl_2_, 10 HEPES, 0.2 Na-GTP, 5 Mg-ATP, with an osmolarity of 285 mOsm/L. These experiments were conducted in transgenic GAD67-GFP C57BL/6 mice to enable fluorescent identification of inhibitory interneurons. Cells were held at their resting membrane potential and drugs were perfused onto the slice for at least 5 min before membrane potential measurements. Membrane potential measurements were taken as averaged baseline potential, excluding any increase in voltage due to action potential discharge.

### 2.3 Extracellular field recordings

Individual slices (350–400 μm) were transferred to an interface recording chamber with a continuous flow rate of 1.5–2 ml/min aCSF saturated with 95% O_2_/5% CO_2_ at 35 °C. Recording electrodes with a 2–5 MΩ resistance were filled with aCSF and placed in the layer V region of the prelimbic cortex. A bipolar metal stimulating electrode was placed perpendicular to layer II/III of the prelimbic cortex.

Field excitatory post synaptic potentials (fEPSPs) were evoked via a 0.05 Hz, 0.1 ms square pulse, generated by a MASTER-8 (A.M.P.I) pulse generator and delivered via a constant current stimulation isolation unit (DS2A, Digitimer). Stimulus intensity that gave 50% maximum response was used for the entire recording. fEPSPs were recorded via an Axoclamp-2A amplifier (Axon Instruments) and digitised at a sample rate of 10 kHz (CED Micro 1401 analogue-digital converter).

For all experiments a 20 min stable fEPSP baseline was recorded ensuring no continuous increase or decrease in response slope. Long term potentiation (LTP) was induced via a high frequency theta burst stimulation (4 bursts 10 s apart, each burst contained 7 trains 140 ms apart, each train contained 4 pulses at 100 Hz.).

### 2.4 Materials

Methyllycaconitine (MLA), donepezil, TTX and 6,7-dinitroquinoxaline-2,3-dione (DNQX) were purchased from Abcam. PNU-120596 and PNU-282987 were provided by Pfizer Inc. USA. All other drugs and compounds were purchased from Sigma Aldrich.

### 2.5 Data analysis

Data were acquired and analysed for both patch clamp and field recordings using WinEDR and WinWCP software (Strathclyde University). fEPSP and eEPSC mean baseline was calculated by averaging the final 5 time points of the baseline before theta burst stimulation or drug application, and used to normalise the data. fEPSP and eEPSC data were excluded if baseline recordings were unstable. For LTP data, average potentiation between 50 and 60 min post theta burst was used for statistical comparison between conditions. Miniature and spontaneous EPSCs and IPSCs were detected using a threshold of 3–7 pA and manually inspected to eliminate false events.

Sample sizes are shown as n = number of recordings (taken from number of individual animals in parentheses). Spontaneous and miniature EPSC and IPSC frequencies and amplitudes were analysed via the nonparametric Kolmogorov–Smirnov test (K–S test) with p values adjusted for multiple comparisons. The same number of events was taken for each cell so as not to skew results towards cells with higher frequencies. Significant differences from control obtained via K–S test are represented as asterisks on histograms and assigned when *p* ≤ 0.01. Excitatory to inhibitory ratios were calculated by dividing the number of sEPSC events/min by the number of sIPSC events/min. Differences were statistically compared by repeated measures one-way ANOVA. All other data were analysed via paired or unpaired Students *t*-tests and significance was assigned when *p* ≤ 0.05.

## 3 Results

### 3.1 α7 nAChRs enhance both excitatory and inhibitory signalling in the prelimbic cortex

Initial experiments in which the selective α7 nAChR agonist PNU-282987 (300 nM) (Hajós et al., 2005) was bath applied to slices of mouse prefrontal cortex did not show any effect on the frequency of spontaneous EPSCs or IPSCs recorded from layer V of the prelimbic cortex (control vs agonist, EPSC: 472 ± 49 and 469 ± 50; IPSC: 1369 ± 178 and 1355 ± 166 events/min respectively, n = 5(3) *p* > 0.05; K–S test, Supplementary Fig. S1). The relatively slow kinetics of bath application of drugs, combined with very rapid agonist-induced desensitization of α7 nAChRs (Papke and Porter Papke, 2002) could have prevented any α7 nAChR activation from occurring or being detected. To overcome this we utilised the selective α7 nAChR type II positive allosteric modulator (PAM) PNU-120596, which potentiates and prolongs α7 nAChR currents (Hurst et al., 2005). PNU-120596 (10 μM) was applied in combination with PNU-282987, to investigate whether increased α7 nAChR activation modulates excitatory and inhibitory neurotransmission within the prelimbic cortex. By repeatedly switching the holding voltage of patch-clamped cells from −60 mV to 0 mV we alternately measured the frequency of spontaneous EPSCs and IPSCs, within the same cell (Semyanov and Kullmann, 2000), in response to the sequential bath application of α7 nAChR drugs.

**Fig. 1:**
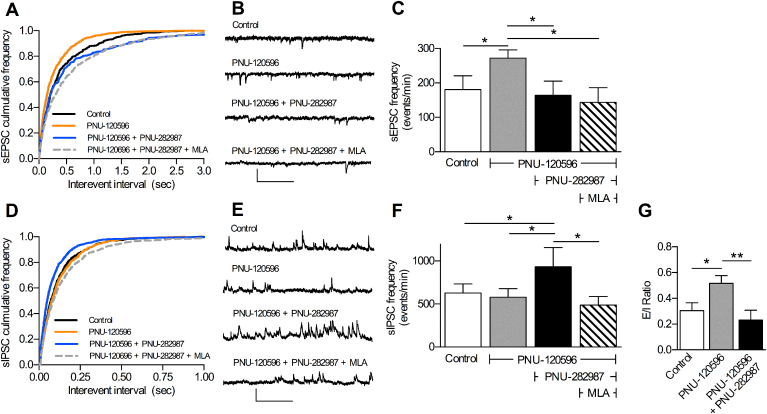
α7 nAChR activation differentially regulates excitatory and inhibitory signalling in the prelimbic cortex. Spontaneous EPSCs (sEPSCs) and IPSCs (sIPSCs) were recorded from the same layer V pyramidal neurons of the prelimbic cortex by switching the holding voltage between −60 mV and 0 mV respectively (see Methods). The α7 nAChR-selective PAM PNU-120596 (10 μM) alone, followed by PAM plus α7 nAChR-selective agonist PNU-282987 (300 nM) were bath applied, before addition of 100 nM MLA. A,D. Cumulative inter-event interval distribution of sEPSCs (A) and sIPSCs (D) in the presence and absence of different drug combinations. B,E. example traces of spontaneous events. C,F. summary of sEPSC (C) and sIPSC (F) frequencies * significantly different, p ≤ 0.001, K–S test based on corresponding cumulative frequency plots n = 7 (4). G. Excitatory/inhibitory (E/I) transmission ratio * significantly different, p ≤ 0.05, ** significantly different, p ≤ 0.01: one-way repeated measures ANOVA with Dunnett's post hoc test, n = 7 (4). Scale bars: 30 pA and 0.5 s.

Bath application of the α7 nAChR PAM PNU-120596 (10 μM) alone significantly enhanced the frequency of spontaneous EPSCs. In contrast to expectations, when the α7 nAChR PAM PNU-120596 and the α7 nAChR agonist PNU-282987 were co-applied, the frequency of spontaneous EPSCs was significantly decreased, compared with PAM alone (Fig. 1A–C). Subsequent application of the α7 nAChR antagonist MLA (100 nM) had no further effect.

Measurement of spontaneous IPSCs from the same neurons revealed substantially different effects of α7 nAChR activation on inhibitory input. PNU-120596 alone did not significantly alter the frequency of spontaneous IPSCs (*p* = 0.394; K–S test), whereas co-application of PNU-120596 and PNU-282987 significantly increased the spontaneous IPSC frequency, compared with control (*p* < 0.001; K–S test) and this increase was reversed on application of MLA (100 nM) (p < 0.001; K–S test) (Fig. 1D–F).

Being able to measure the excitatory and inhibitory signalling onto the same neuron within an intact network enables the determination of the excitatory to inhibitory neurotransmission ratio (E/I ratio), which highlights the overall network consequence of α7 nAChR modulation (Semyanov and Kullmann, 2000). Upon application of PNU-120596 alone, the E/I ratio significantly increased (Fig. 1G), showing an overall net increase in excitatory neurotransmission. Upon co-application of PNU-120596 and PNU-282987 this enhancement in the E/I ratio was significantly reversed (Fig. 1G) consistent with a switch from overall enhanced excitatory drive to a predominantly inhibitory drive.

### 3.2 α7 nAChRs are located on GABA interneurons and glutamate terminals

**Fig. 2:**
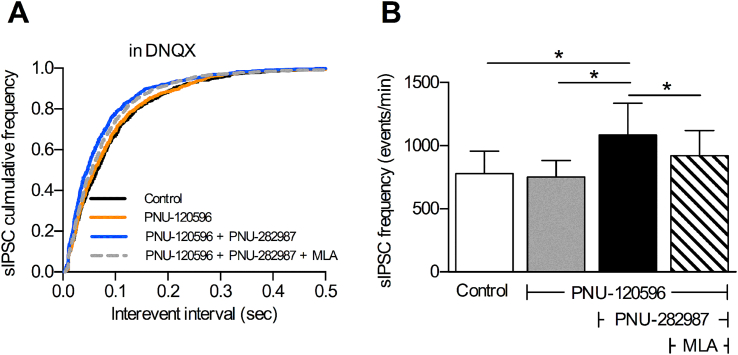
α7 nAChR-induced increase in spontaneous IPSC frequency is independent of glutamate signalling. Spontaneous IPSCs (sIPSCs) were recorded in the presence of the AMPA receptor antagonist DNQX. A. Cumulative inter-event interval distributions, showing the change in sIPSC frequency in the presence of DNQX (10 μM) after bath application of α7 nAChR PAM (PNU-120596; 10 μM) alone and with α7 nAChR agonist (PNU-282987; 300 nM), in the absence then presence of 100 nM MLA. B. Summary histogram of sIPSC frequencies.* Significantly different, p ≤ 0.001, K–S test based on cumulative frequency plots shown in A; n = 6 (4).

The increase in inhibitory input recorded following co-application of α7 nAChR PAM and agonist could reflect increased glutamate release onto the GABAergic interneurons. This possibility was ruled out by the inability of the AMPA receptor antagonist DNQX (10 μM) to prevent the increase in spontaneous IPSC frequency produced by co-application of PNU-120596 and PNU-282987. In the presence of DNQX, co-application of PNU-120596 and PNU-282987 still caused a significant increase in spontaneous IPSC frequency, which was significantly reduced by MLA (100 nM) (Fig. 2).

**Fig. 3:**
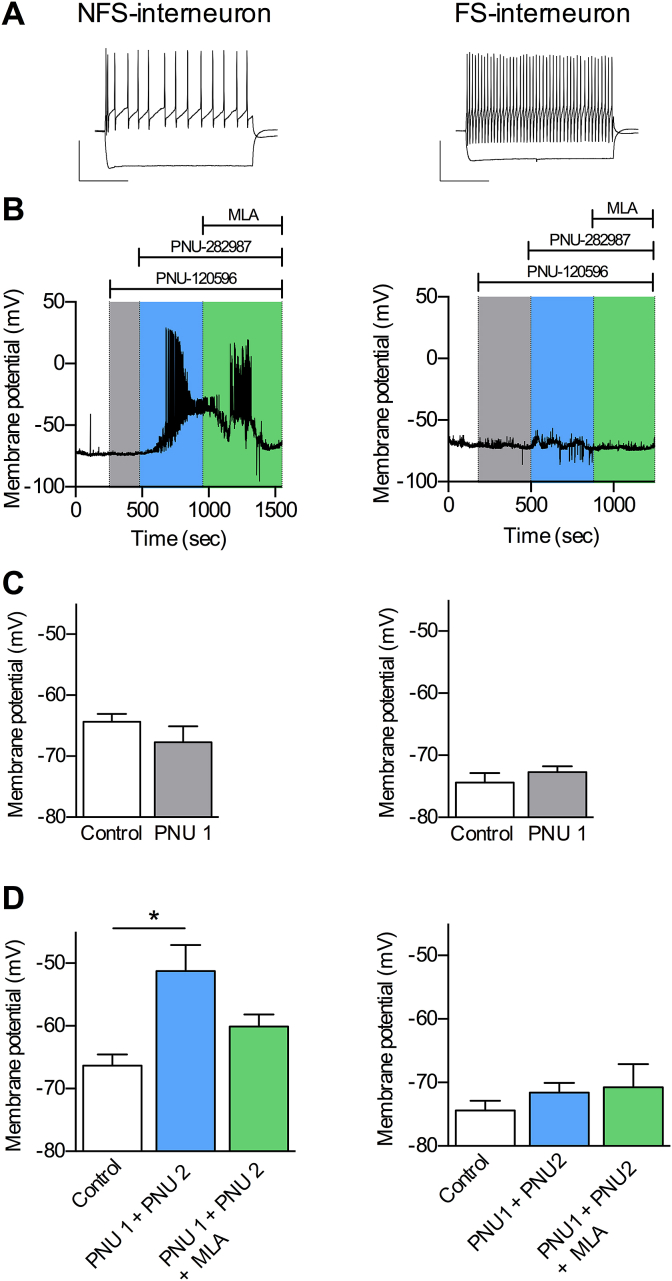
α7 nAChR activation but not positive allosteric modulation directly depolarises layer V inhibitory interneurons. Changes in the membrane potential of layer V non-fast spiking (NFS) and fast spiking (FS) inhibitory interneurons were recorded in response to α7 nAChR modulation activation and inhibition. A. Spiking profile of layer V NFS (left) and FS (right) interneurons in response to a 300 ms current injection of +150 pA or −150 pA. Scale bars 50 mV and 100 ms. B. Representative current clamp recording from NFS (left) and FS (right) showing the changes in membrane potential in response to 10 μM PNU-120596 (‘PNU 1’), co-application of PNU-120596 and 300 nMPNU-282987 (‘PNU1 + PNU 2’), and addition of 100 nM MLA. C. Averaged membrane potential in response to 10 μM PNU-120596 in NFS (left; n = 3) and FS (right; n = 5) interneurons; recordings from 3 animals (C), and in response to co-application of PNU-120596 and 300 nM PNU-282987 followed by addition of 100 nM MLA in NFS (left; n = 5) and FS (right; n = 5); recordings from 3 animals (D). * significantly different from control, p ≤ 0.05, one-way repeated measures ANOVA with Dunnett's post hoc test.

Having excluded increased excitatory input as a mediator of α7 nAChR-elicited increases in inhibitory signalling, we hypothesised that α7 nAChRs residing on the GABA interneurons themselves were responsible. We conducted whole cell current clamp recordings in layer V fast spiking and non-fast spiking inhibitory interneurons in slices taken from GAD67-GFP knock-in mice to permit visual identification of GABAergic interneurons (Tamamaki et al., 2003). The fast spiking and non-fast spiking inhibitory interneurons were identified by their characteristic properties (Supplementary Fig. S2), for example non-fast spiking interneurons displayed a lower frequency of action potentials following current injection compared with fast spiking interneurons (Fig. 3A). Application of PAM alone onto 8 interneurons from 3 GAD67-GFP mice revealed no depolarisation of either cell type (Fig. 3B, C). In contrast we observed a significant depolarisation of non-fast spiking, but not fast spiking, interneurons in response to co-application of PNU-120596 and PNU-282987, and this was blocked by MLA (Fig. 3B, D). In the majority of non-fast spiking neurons, this depolarisation was sufficient to induce action potential discharge (see Fig. 3B), and provides evidence that α7 nAChRs residing on cell bodies of non-fast spiking inhibitory interneurons can directly alter the levels of inhibitory signaling within the prelimbic cortex.

**Fig. 4:**
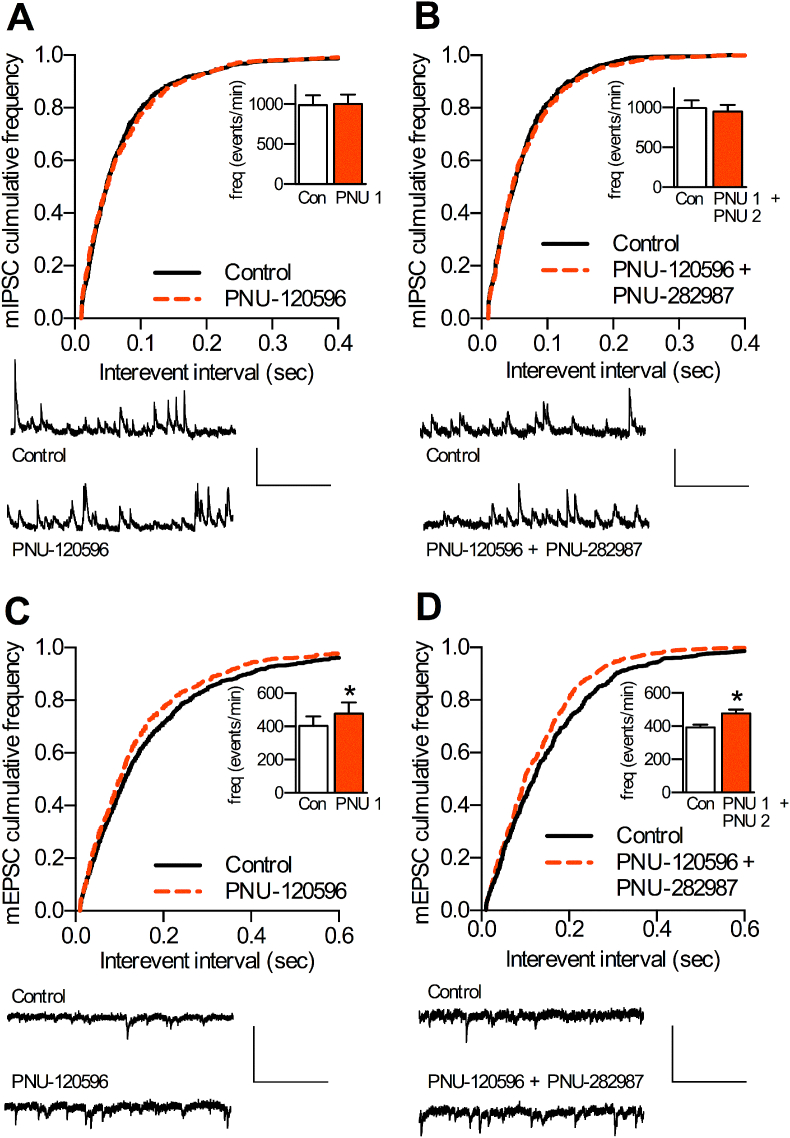
Effect of α7 nAChR activation on miniature EPSCs and IPSCs. Miniature IPSC (mIPSC) and EPSC (mEPSC) frequencies were measured in layer V pyramidal neurons of the prelimbic cortex in the presence of tetrodotoxin (1 μM) to block axonal conduction. Cumulative distribution and summary histograms (inserts) of mIPSC (A,B) and mEPSC (C,D) frequencies in the presence or absence of PNU-120596 (10 μM) alone (‘PNU 1’; n = 11 (9)) (A,C) or PNU-120596 (10 μM) co-applied with PNU-282987 (300 nM) (‘PNU 1 + PNU 2’; n = 5 (4)) (B), or PNU-282987 (30 nM) (‘PNU 1 + PNU 2’; n = 3 (3)) (D). Representative traces are shown beneath each panel. * significantly different from control, p ≤ 0.001, K–S test based on corresponding cumulative frequency plot. Scale bars: 30 pA and 0.5 s.

To determine whether α7 nAChRs are located on nerve terminals of GABAergic interneurons, miniature IPSCs were recorded from layer V pyramidal neurons, in the presence of tetrodotoxin (1 μM). PNU-120596 alone and co-application of PNU-120596 with PNU-282987 failed to alter either the frequency or amplitude of miniature IPSCs (Fig. 4A, B). This result provides no evidence for presynaptic α7 nAChRs on mPFC interneurons, and we conclude that α7 nAChRs are located on the cell bodies (of fast-spiking interneurons) but not nerve terminals of interneurons, in agreement with previous studies (Couey et al., 2007, Aracri et al., 2010, Poorthuis et al., 2012), and see Fig. 3.

When interneuron action potential firing was abolished by the use of tetrodotoxin α7 nAChR activation no longer affected inhibitory input. This strategy enabled us to observe the effects of presynaptic α7 nAChRs on excitatory transmission in isolation. In contrast to miniature IPSCs, application of the α7 nAChR PAM (PNU-120596) significantly increased miniature EPSC frequency (Fig. 4C) with no effect on miniature EPSC amplitude (8.3 ± 0.4 to 7.9 ± 0.31 pA, n = 11 (9) *p* > 0.05; K–S test). These data suggest that α7 nAChRs are located on nerve terminals of afferent glutamatergic inputs.

Co-application of PNU-120596 (10 μM) and PNU-282987 (300 nM) in the presence of TTX resulted in a substantial fluctuation of membrane current in recorded cells (Supplementary Fig. S3), presumably due to high levels of α7 nAChR-driven glutamatergic input in the absence of inhibitory modulation. Application of a lower (submaximal) concentration of PNU-282987 (30 nM) together with PNU-120596 (10 μM) produced stable recordings that revealed a significant increase in the frequency of miniature EPSCs (Fig. 4D) with no change in miniature EPSC amplitude (8.3 ± 0.6 to 8.0 ± 0.3 pA, n = 3 (3) *p* > 0.05; K–S test). Together these data suggest that exogenous activation of α7 nAChRs with PNU-282982 and PNU-120596 is capable of enhancing presynaptic glutamate release, but this effect is attenuated via α7 nAChR activation of inhibitory interneurons in a functionally intact network as observed in spontaneous EPSCs (Fig. 1*A-C*).

### 3.3 Acetylcholinesterase inhibition enhances PNU-120596-induced increases in EPSCs

The bidirectional effect of α7 nAChR activation on layer V pyramidal neurons of the prelimbic cortex reflects activation of all of the α7 nAChRs within the slice, through the combined action of a selective α7 nAChR agonist and PAM, leading to an increase in inhibitory drive onto layer V pyramidal neurons, whereas application of the α7 nAChR PAM alone selectively enhances excitatory drive onto layer V neurons. This difference could reflect a different spatial or temporal relationship between cholinergic afferents and glutamatergic nerve terminals, such that local ACh preferentially targets presynaptic α7 nAChRs that modulate glutamate release, rather than α7 nAChRs on the cell bodies of GABAergic interneurons.

**Fig. 5:**
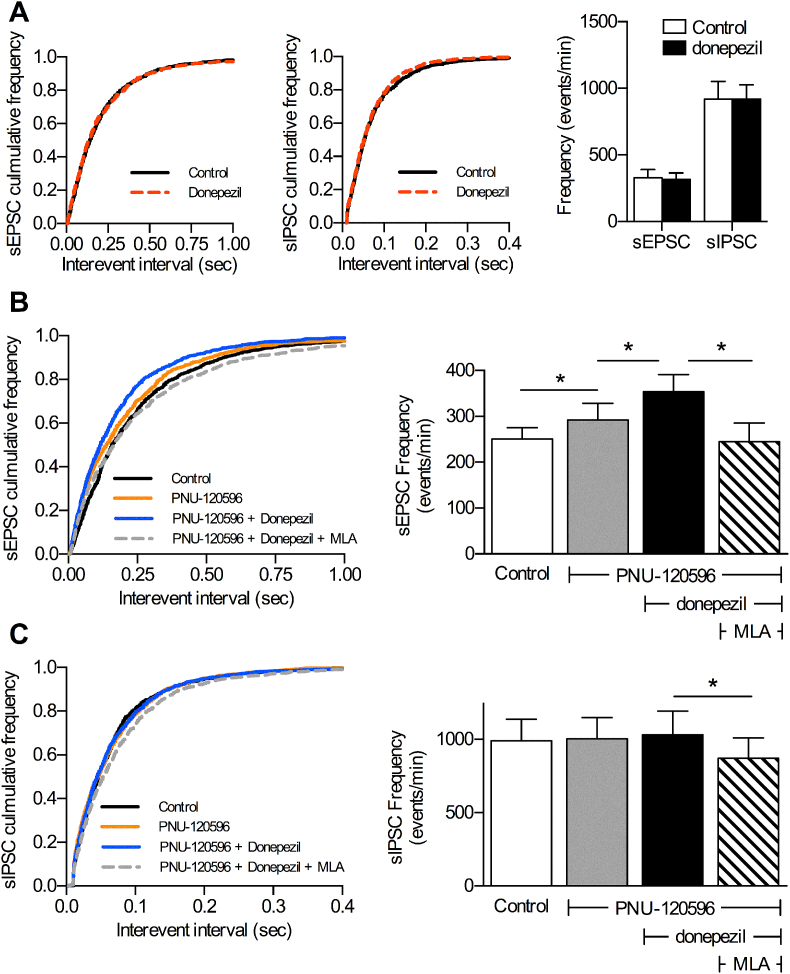
Enhancing endogenous acetylcholine selectively potentiates excitatory signalling. Spontaneous EPSCs (sEPSCs) and IPSCs (sIPSCs) were recorded from layer V pyramidal neurons in the presence and absence of donepezil (10 μM) to block acetylcholinesterase activity. A. Cumulative inter-event interval distributions and summary histogram of sEPSC and sIPSC frequencies confirm that donepezil had no effect on either sEPSC or sIPSC frequency; n = 8 (5). B,C Cumulative inter-event interval distributions and summary histogram of sEPSCs (B) and sIPSCs (C) in the presence and absence of PNU-120596 (10 μM), donepezil (10 μM), and MLA (100 nM), n = 6 (6). * significantly different, p ≤ 0.001, K–S test based on corresponding cumulative frequency plot.

To test this hypothesis we further enhanced the levels of endogenous ACh by preventing its enzymatic breakdown, using the acetylcholinesterase inhibitor donepezil. Donepezil alone (10 μM) did not significantly alter the frequency of spontaneous EPSCs or IPSCs (Fig. 5A). However, in the presence of the α7 nAChR PAM PNU-120596 (10 μM), donepezil significantly increased the spontaneous EPSC frequency, compared to both control and to PNU-120596 alone (Fig. 5B). This enhancement was reversed by MLA (100 nM). In contrast, donepezil did not alter the spontaneous IPSC frequency, with or without PNU-120596 (Fig. 5C). These data support the hypothesis that endogenous ACh release targets α7 nAChRs that selectively enhance excitatory neurotransmission, and not inhibitory neurotransmission, in layer V of the prelimbic cortex.

MLA was also applied alone to investigate the level of tonic background receptor activation. In these experiments MLA alone caused a slight overall decrease in sEPSC frequency. A clear decrease in frequency was seen in 3 out of 6 neurons (Overall: 313 ± 34 (control) to 275 ± 34 (+MLA) events/min, n = 6 (4) *p* = 0.04; K–S test). No effect of MLA was seen on sIPSC frequency (691 ± 99 to 714 ± 97 events/min, n = 6(4) *p* = 0.85; K–S test) (Supplementary Fig. S4). This is consistent with the hypothesis that tonic ACh preferentially activates α7 nAChRs on glutamatergic nerve terminals, although the magnitude of effect of MLA was less than that seen when α7 nAChRs were further activated with the α7 PAM.

### 3.4 Both antagonism and activation of α7 nAChRs decrease evoked glutamate release

**Fig. 6:**
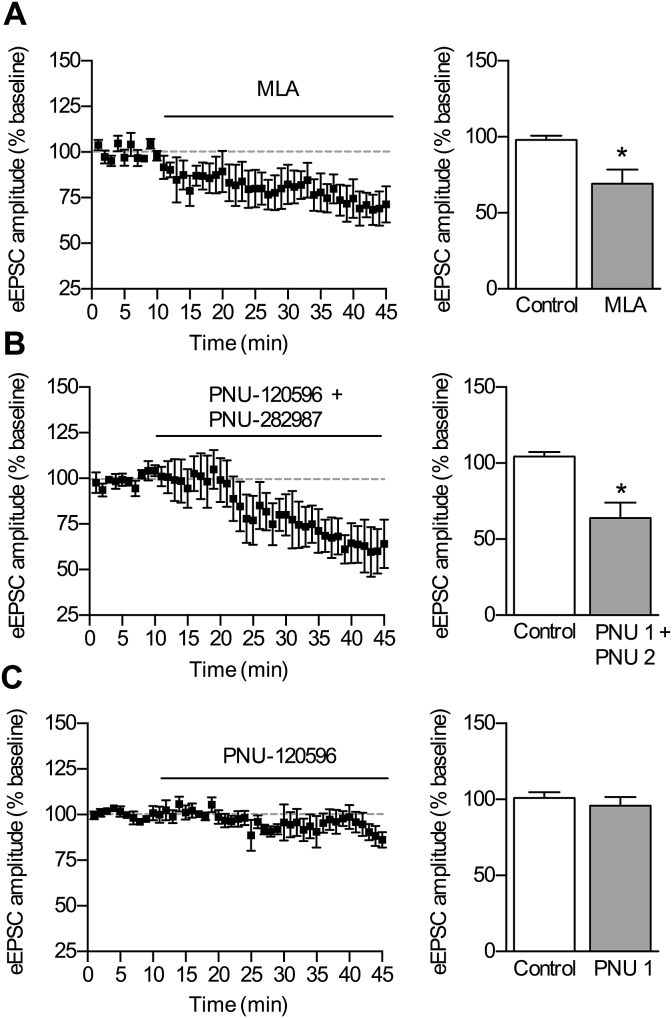
Both antagonism and activation of α7 nAChRs decreases evoked EPSC amplitudes. EPSCs were evoked in layer V pyramidal neurons by stimulation of distal dendrites in layers II/III of the prelimbic cortex. The amplitude of these EPSCs was measured before and after bath application of (A) MLA (100 nM), n = 4 (4), (B) co-application of the α7 nAChR PAM PNU-120596; (10 μM) and α7 nAChR agonist PNU-282987 (300 nM), n = 5 (5), and (C) α7 nAChR 10 μM PAM alone, n = 4 (4). Time-courses show amplitude of evoked potentials as a % of mean control potentials that were collected for 20 min in the absence of drugs; histograms show averaged data at time points 10 min (control) and 40 min (drug). * significantly different from control, p ≤ 0.05, paired t-test.

To further investigate the role of α7 nAChRs in excitation within the prelimbic cortex we examined the receptors’ role in modulating evoked neurotransmitter release. Electrically stimulating layers II/III of the prelimbic cortex and recording from pyramidal neurons in layer V held at −60 mV, in the absence of GABA_A_ receptor blockade, resulted in a DNQX-sensitive glutamatergic response. Upon prolonged bath application of the α7 nAChR antagonist MLA (100 nM), the amplitudes of evoked EPSCs significantly decreased (Fig. 6A). Similarly, co-application of α7 nAChR PAM PNU-120596 (10 μM) and α7 nAChR agonist PNU-282987 (300 nM) also significantly reduced evoked EPSC amplitudes (Fig. 6B). However, application of PNU-120596 alone failed to alter evoked EPSC amplitudes (Fig. 6C). The quantitative difference of the effect of PAM on spontaneous and evoked glutamate responses could reflect their differential modulation by α7 nAChRs.

### 3.5 α7 nAChRs modulate LTP in the prelimbic cortex

The evidence for the bidirectional control that α7 nAChRs exert on spontaneous glutamate and GABA release, as well as their ability to affect pronounced glutamate release, suggests that α7 nAChRs could also play a role in modulating more complex network activity. To explore this possibility, we assessed the ability of α7 nAChR modulation to influence long term potentiation (LTP) within the prelimbic cortex.

**Fig. 7:**
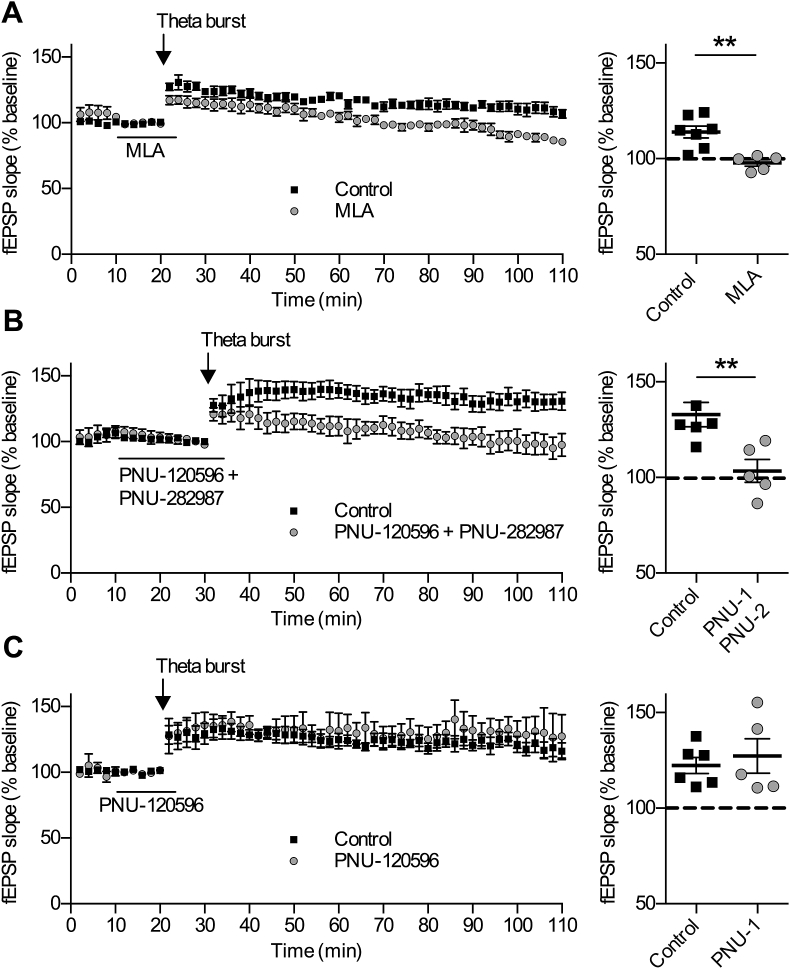
Both antagonism and activation of α7 nAChRs inhibit long-term potentiation in layer V of the prelimbic cortex. Field EPSPs (fEPSPs) were recorded from prelimbic layer V; long-term potentiation was induced via a theta burst stimulation in layer II/III. Recordings were made in the absence (control n = 7 (7)) and presence of bath applied MLA (100 nM; n = 5 (5)) (A), α7 nAChR PAM PNU-120596 (10 μM) and agonist PNU-282987 (300 nM) (control; n = 6 (6); drug n = 5 (5)) (B), and 10 μM PNU-120596 alone (control n = 6 (6); drug n = 5 (5) (C). MLA and PNU-120596 were bath applied for 10 min before and during theta-burst stimulation whereas PNU-120596 + PNU-282987 co-application was bath applied for 20 min. Histogram (right) shows % LTP potentiation from baseline taken 50–60 min post theta burst for each slice. Both MLA and co-application of PNU-120596 and PNU-282987 significantly reduced the levels of LTP from control, p ≤ 0.05, t-test.

Extracellular field recordings were conducted to measure the level of theta burst-induced LTP in the presence of different α7 nAChR ligands, to promote or inhibit α7 nAChR activity. Firstly, MLA was used to reveal any intrinsic α7 nAChR contribution arising from endogenous ACh. In this case MLA was predicted to reduce the level of LTP by decreasing α7 nAChR-enhanced glutamate signaling. Indeed, application of MLA (100 nM) 10 min before and during a theta burst stimulation produced a significant reduction in the level of LTP (Fig. 7A). Confirmation that this was α7 nAChR-mediated was provided by the pseudo-irreversible α7 nAChR antagonist α-bungarotoxin (α-BGT; 300 nM), which produced a comparable decrease in LTP (control: 165 ± 19% baseline, n = 4 (4), α-BGT: 120 ± 5% baseline, n = 5 (5); p < 0.05; t-test 60 min post theta-burst; Supplementary Fig. S5.)

Combined application of both PAM (PNU-120596; 10 μM) and agonist (PNU-282987; 300 nM), 20 min before and during a theta burst stimulation also resulted in a significant reduction in the level of LTP (Fig. 7B). This is consistent with an enhancement of inhibitory signaling, as seen in the measurements of spontaneous IPSCs and the reduction in evoked glutamate in response to α7 nAChR PAM and agonist application.

In view of the inability of α7 nAChR PAM alone to alter the levels of evoked glutamate (Fig. 6C), we predicted that positive allosteric modulation of the α7 nAChR should not alter the levels of theta burst LTP. Indeed, we found that LTP recorded in the presence of PNU-120596 (10 μM) was indistinguishable from that in control slices (Fig. 7C).

## 4 Discussion

We show that α7 nAChRs play a complex bidirectional role in mediating network excitability in the prelimbic cortex. Enhancing cholinergic tone with the α7 nAChR PAM PNU-120596 increases overall excitation whereas global activation of α7 nAChRs with a combination of agonist (PNU-282987) and PAM enhances overall inhibition. Both global activation (by PAM and agonist) *and* inhibition (by the α7 nAChR antagonist MLA) of α7 nAChRs inhibited induction of theta-burst-induced LTP in the prelimbic cortex, reflecting the differential effects of α7 nAChRs on excitation and inhibition.

### 4.1 Bi-directional effects of α7 nAChR activation

It is well recognised that α7 nAChRs can regulate both excitatory and inhibitory signalling in the brain (Griguoli and Cherubini, 2012, Yakel, 2013, Hedrick and Waters, 2015). In the PFC, layer V pyramidal neurons are excited by nAChRs that enhance glutamatergic inputs (hitherto attributed to β2* nAChRs (Lambe et al., 2003)) and nAChRs also increase inhibition to layer V pyramidal neurons (Couey et al., 2007). Recording spontaneous PSCs alternately at 0 mV and −60 mV (corresponding to the estimated reversal potentials of ionotropic glutamate and GABA_A_ receptors, respectively (Semyanov and Kullmann, 2000)) enabled the recording of inhibitory and excitatory events in the same cell, without pharmacological blockade of the preparation. This has revealed that individual layer V pyramidal neurons are subject to excitatory and inhibitory inputs that are both enhanced by α7 nAChRs. By avoiding the need for blockers of GABA_A_ or glutamate receptors, the net effect of α7 nAChRs’ modulatory influences on evoked responses and synaptic plasticity could be evaluated.

The ability of α7 nAChR-selective PAM plus agonist to depolarise inhibitory interneurons and increase spontaneous IPSCs, in a TTX-sensitive and DNQX-independent manner, is consistent with the well documented evidence for somatic α7 nAChRs on GABAergic interneurons in the PFC (Couey et al., 2007, Aracri et al., 2010). We show that within the prelimbic layer V, non-fast spiking inhibitory interneurons undergo a more pronounced depolarisation to α7 nAChR activation compared to fast spiking interneurons. This could be attributed to the differential expression of α7 nAChRs on different interneuron subtypes throughout cortical layers as previously shown (Poorthuis et al., 2012). In addition to functional studies, immunoreactivity attributed to α7 nAChRs has been localised to GABAergic dendritic shafts and somata in guinea pig medial PFC (Lubin et al., 1999) providing some ultrastructural evidence for this association.

In contrast, α7 nAChRs on glutamatergic boutons in the prelimbic cortex are inferred from the ability of the α7 nAChR PAM PNU-120596 to enhance spontaneous EPSC frequency in a manner that was insensitive to TTX, as seen by the PAM-induced increase in miniature EPSCs (Fig. 4). There is functional and ultrastructural evidence for presynaptic α7 nAChRs on hippocampal mossy fibre terminals (Gray et al., 1996, Sharma et al., 2008, Cheng and Yakel, 2014) and glutamatergic inputs to the ventral tegmental area (Jones and Wonnacott, 2004, Good and Lupica, 2009, Garzón et al., 2013). Evidence for presynaptic α7 nAChRs in the PFC is limited: local infusion of an α7 nAChR agonists into the rat PFC have been shown to transiently increased glutamate release (Konradsson-Geuken et al., 2009, Bortz et al., 2013), consistent with the demonstration of functional α7 nAChRs on excitatory amino acid nerve terminals (Dickinson et al., 2008).

Nicotine or ACh provokes a large excitation of layer V pyramidal neurons in the PFC, but this has previously been attributed to α4β2* nAChRs on thalamo-cortical terminals (Lambe et al., 2003, Couey et al., 2007, Poorthuis et al., 2013). In the present study we specifically examined α7 nAChRs by utilising subtype-selective pharmacological tools. This approach has revealed a presynaptic action of α7 nAChRs that may have been either masked, in the presence of a larger α4β2* nAChR-mediated response, or absent due to desensitization in previous studies. The ability of MLA to inhibit theta-burst stimulated LTP (Fig. 7) argues for α7 nAChRs making a physiological contribution to overall activity in the PFC. Interestingly, we saw no somatic currents induced by α7 nAChRs in layer V pyramidal cells, unlike some previous reports (Poorthuis et al., 2013), but see (Hedrick and Waters, 2015).

### 4.2 Cholinergic signalling mediated by α7 nAChRs in the prelimbic cortex

The α7 nAChR PAM PNU-120596, applied in the absence and presence of exogenous agonist, revealed the contribution of endogenous ACh to excitatory, but not inhibitory, signalling. The PAM alone elicited MLA-sensitive increases in spontaneous EPSC frequency, and additional enhancement by the AChE inhibitor donepezil corroborates a role for endogenous ACh in modulating glutamate release. Choline, the breakdown product of ACh when metabolised by AChE, is also an α7 nAChR agonist (Alkondon et al., 1997, Innocent et al., 2008). Our findings with donepezil, however, indicate that the endogenous α7 nAChR activation observed is via ACh, not choline.

**Fig. 8:**
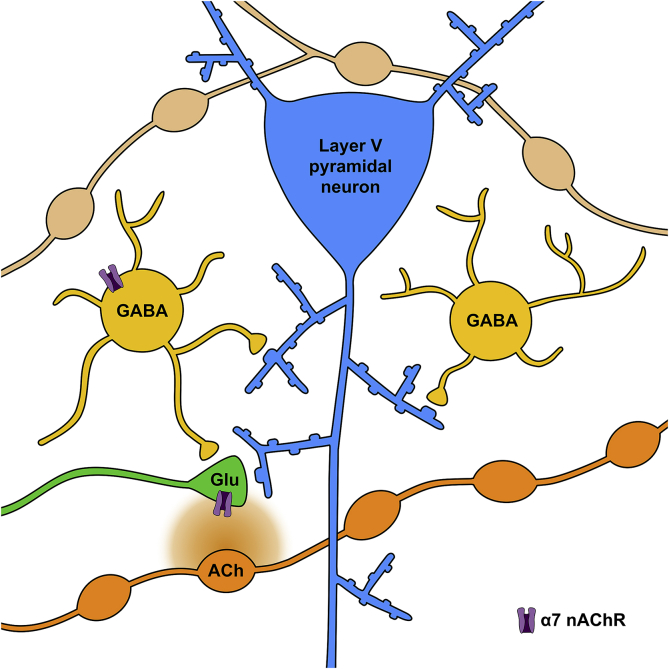
Layer V pyramidal neurons are dynamically controlled by α7 nAChRs. A model illustrating the potential locations of α7 nAChRs and the interplay between glutamatergic, GABAergic and cholinergic systems in layer V of the prelimbic cortex. α7 nAChRs are shown on the terminal of a glutamatergic afferent (green) and on cell bodies of inhibitory interneurons (yellow). Thus α7 nAChRs are able to influence both the excitability and inhibition of layer V pyramidal neurons (blue). Tonic endogenous ACh selectively targets α7 nAChRs on glutamatergic terminals, suggesting close proximity to tonically active en passant varicosities (orange). In contrast, GABAergic interneurons bearing α7 nAChRs are either more distant from cholinergic fibres, or localised close to tonically inactive varicosities (brown). (For interpretation of the references to colour in this figure legend, the reader is referred to the web version of this article.).

The efficacy of the PAM alone in increasing EPSCs suggests that, in the slice, sufficient ACh persists in the vicinity of glutamatergic boutons to activate α7 nAChRs when the receptors are sensitised by the PAM; this may signify a local, tonic release of ACh. In contrast the inability of the PAM alone, but the requirement for both PAM and agonist, to elicit a depolarisation of inhibitory interneurons and an increase in layer V pyramidal neuron IPSCs suggests that inhibitory neurons are subject to a distinct mode of cholinergic signalling. This may rely on activity-dependent ACh release from a population of cholinergic fibres that form direct synaptic connections with α7 nAChR-expressing inhibitory interneurons (Bennett et al., 2012). Alternatively, it could reflect a different spatial relationship such that tonically released ACh is insufficiently available to α7 nAChRs on GABAergic interneurons (Fig. 8). We cannot presently distinguish between these possibilities. Cholinergic innervation from the basal forebrain is widely distributed through the cortical layers, apart from layer IV where it is less dense (Aracri et al., 2010, Bloem et al., 2014b). Projections from the basal forebrain are physiologically heterogeneous, with respect to conduction velocity and spontaneous activity (Aston-Jones et al., 1985), and the predominant varicose pattern of innervation is consistent with a paracrine delivery of ACh (Duffy et al., 2009, Aracri et al., 2010). In addition, sparse, intrinsic cholinergic interneurons are also present in the cortex, but these predominate in layers 2 and 3 (Engelhardt et al., 2007).

Interestingly, although there was sufficient basal cholinergic tone for the PAM alone to enhance glutamate release onto pyramidal cells, this effect was attenuated by co-administration of PAM and agonist (Fig. 1A–C). This suggests that GABAergic interneurons may provide inhibitory input to glutamatergic terminals (Fig. 8), such that global activation of α7 nAChRs can overcome the direct excitatory effect of α7 nAChRs on glutamatergic terminals.

This finding is consistent with the reduced levels of evoked EPSCs observed in response to co-administration of the α7 PAM plus agonist (Fig. 6B). Surprisingly, evoked glutamate levels were not enhanced upon α7 PAM administration alone. This finding may be explained by the relative levels of glutamate and ACh released under evoked versus basal conditions. Evoked release would be expected to elevate cholinergic tone that may produce a near maximal effect at glutamatergic nerve terminals, limiting any potentiation with a PAM. If this is correct then with elevated levels of ACh during evoked experiments we would expect to be able to inhibit its effect with MLA, as seen in Fig. 6A. Whereas with lower tonic levels of ACh during spontaneous experiments we might expect not to see a pronounced reduction in glutamate release as seen by only a modest reduction in spontaneous glutamate release upon MLA application (Supplementary Fig. S4).

### 4.3 Roles of α7 nAChRs in integrating network activity

The PFC in general, including the prelimbic cortex, has attracted considerable attention because of its importance for executive function and attentional processes. α7 nAChRs in this brain region have been studied as a possible novel target for cognitive and executive-control disorders such as schizophrenia, Alzheimer's disease, attention-deficit hyperactivity disorder and addiction states (Pandya and Yakel, 2013, Poorthuis and Mansvelder, 2013, Wallace and Bertrand, 2013). Our finding that α7 nAChRs play a bidirectional role in network activity in the prelimbic cortex point to a more complex function for α7 nAChRs in this brain region than previously recognised.

The differential effects of PAM and agonist on excitatory and inhibitory signalling provides a means of discriminating their contributions to network activity manifest in evoked responses and synaptic plasticity. Synaptic plasticity is widely recognised as a neuronal correlate of learning and memory, and α7 nAChRs have been shown to modulate synaptic plasticity. This has been most extensively studied in the hippocampus, where activation of α7 nAChRs enhances LTP induction (Welsby et al., 2006, Lagostena et al., 2008) and LTP induction is attenuated in α7 nAChR knockout mice, or by α7 nAChR antagonists (Welsby et al., 2006, Gu and Yakel, 2011). The dual action of α7 nAChRs on plasticity in the prelimbic cortex also resonate with similar mechanisms in the hippocampus (Ji et al., 2001, Gu and Yakel, 2011). Interestingly, the enhancing effect of the α7 nAChR partial agonist, S 24795, on hippocampal LTP was only seen at lower concentrations; higher concentrations reduced LTP induction (Lagostena et al., 2008). We see similar biphasic actions of α7 nAChRs, depending on the level of receptor activation, on LTP in the prelimbic cortex (Fig. 7). These data provide evidence that α7 nAChRs enhance LTP is via actions at glutamatergic nerve terminals, whereas their inhibitory effect on LTP is via actions at GABAergic interneurons. Previous studies have also shown that nAChRs on inhibitory interneurons influence spike-timing dependent plasticity in the mPFC (Couey et al., 2007). However, these findings were largely attributed to heteromeric nAChRs whereas the present study reveals that homomeric α7 nAChRs can similarly alter plasticity. Complementary roles for β2-containing and α7 nAChRs have also been reported with respect to the modulation of network activity in mouse barrel cortex (Sigalas et al., 2015).

Within the mPFC network, multiple nicotinic and muscarinic AChRs are expressed on distinct network components across multiple cortical layers (Vidal and Changeux, 1993, Poorthuis et al., 2012), suggesting individual ACh receptor subtypes may have complex roles in regulating the cortical network (Parikh et al., 2010). This focussed pharmacological study highlights a new complexity in how α7 nAChRs within this system can bidirectionally influence network activity. They may complement heteromeric nAChRs by differences in subcellular localisation: preterminal α4β2 nAChRs have been inferred on thalamocortical glutamatergic inputs (Lambe et al., 2003), whereas the present studies indicate that α7 nAChRs reside on nerve terminals of glutamatergic inputs.

In addition to differences in spatial expression of multiple ACh receptor subtypes, the different modes of ACh release are increasingly recognised as critical regulators of attentional processes (Sarter et al., 2009). We show that tonic ACh release, acting through α7 nAChRs, can directly modulate excitation whilst elevated ACh signalling activates α7 nAChRs to regulate inhibitory interneuron activity. Understanding how ACh orchestrates network activity through these nAChRs will be critical to understanding the role of ACh in mediating cognitive processes (Bloem et al., 2014a). Our findings suggest that α7 and α4β2 receptors need to be considered together to better understand the functional consequences of ACh release in the mPFC.

In conclusion, we demonstrate that α7 nAChRs in the prelimbic region of the mouse prefrontal cortex exert a bidirectional effect on network excitability. Basal cholinergic tone preferentially activates α7 nAChRs located on glutamatergic nerve terminals and has an overall excitatory effect on pyramidal output cells, whereas global activation of α7 nAChRs leads to enhanced GABAergic input to both pyramidal cells and glutamatergic nerve terminals. These effects result in the seemingly contradictory finding that both inhibition and global activation of α7 nAChRs can inhibit LTP induction in this brain region. α7 nAChRs have attracted interest as potential therapeutic targets for cognitive and attentional disorders such as schizophrenia, Alzheimer's disease and autism (Bertrand et al., 2015). Our findings indicate that differences in the manner and extent by which α7 nAChRs are activated can have different functional and physiological consequences, and highlights the challenge for designing effective drug treatments targeted at α7 nAChRs.

## 5 Conclusions

The prelimbic cortex plays a key role in attention, executive control and decision-making. Outputs reflect the balance of excitatory and inhibitory systems, which are modulated by transmitters like acetylcholine. We investigated how the contributions of acetylcholine acting at α7 nicotinic acetylcholine receptors (nAChRs) alter activity in the prelimbic cortex. Activation of α7 nAChRs increased both excitatory and inhibitory signaling, but with differing sensitivities to acetylcholine. Enhancing available acetylcholine results in overall excitation, whereas global activation of α7 nAChRs with an exogenous agonist results in overall inhibition. These findings are mirrored by effects on synaptic plasticity, where both antagonism and global activation of α7 nAChRs decrease long-term potentiation, highlighting a complex bidirectional role for α7 nAChRs in the prelimbic cortex.
