# Supplementary material for: Integration of inhibitory and excitatory effects of α7 nicotinic acetylcholine receptor activation in the prelimbic cortex regulates network activity and plasticity
Source: Neuropharmacology. 2016 Jun;105:618–29. doi: 10.1016/j.neuropharm.2016.02.028 (PMC4881417; doi:10.1016/j.neuropharm.2016.02.028)
Supplement: Supplementary file 1 [file mmc1.docx]

Udakis et al - Supplementary materials


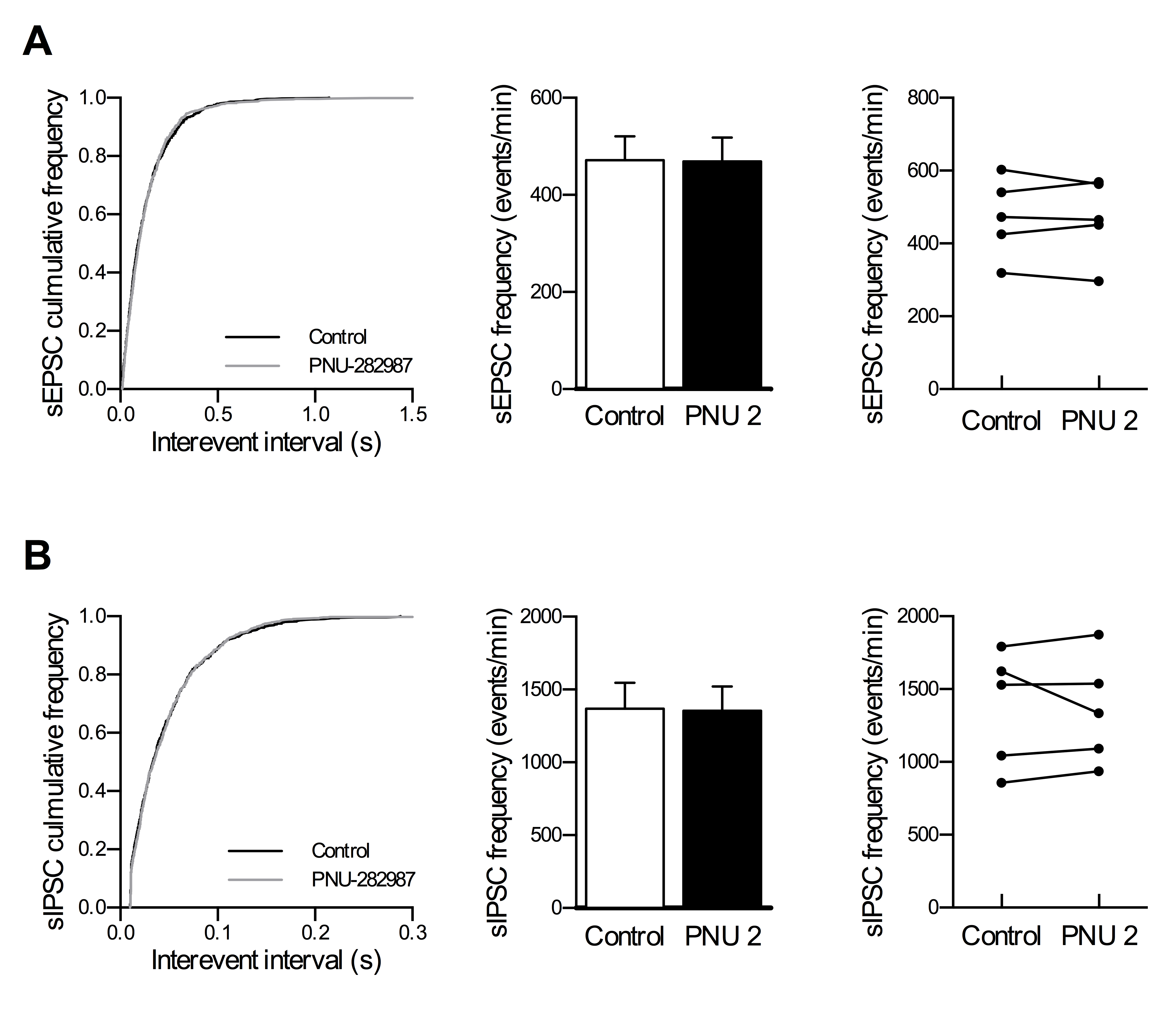


**Figure S1. PNU-282987 application alone results in no significant increase in either sEPSCs or IPSCs**

Spontaneous excitatory (sEPSC) and inhibitory (sIPSC) post synaptic current frequency was measured in layer V pyramidal neurons in the presence and absence of α7 nAChR agonist PNU-282987. Cumulative distribution, summary histogram and individual cell frequencies to application of 300 nM PNU-282987 (‘PNU 2’) for sEPSCs (**A**) and sIPSCs (**B**) (n = 5; *p* > 0.01, K-S test).

**
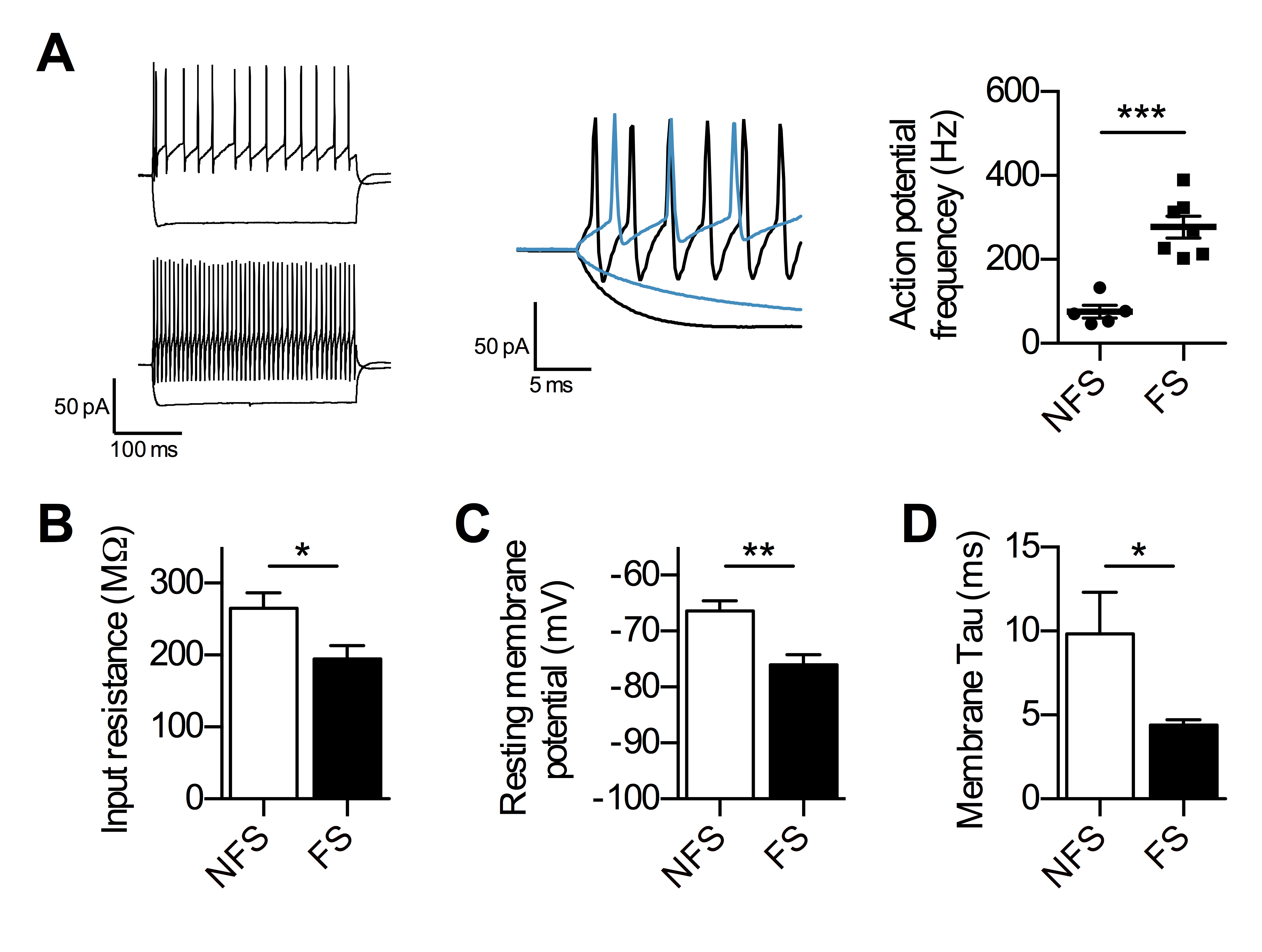
**

**Figure S2. Characterisation of non-fast spiking and fast spiking layer V inhibitory interneurons.**

Current clamp recordings were made from interneurons in layer V of the prelimbic cortex from brain slices of GAD67-GFP transgenic mice. Interneurons were characterised as being fast spiking or non-fast spiking, based on their membrane properties in response to a series of depolarising and hyperpolarising current steps. Non-fast spiking interneurons (upper left trace; blue trace on right) possessed a significantly lower action potential firing rate compared to fast spiking interneurons (lower left trace; black trace on right) during a 300ms 150 pA current step (**A**). Fast spiking interneurons (FS) possessed a significantly lower input resistance (**B**), resting membrane potential (**C**) and membrane constant (**D**) compared to non-fast spiking interneurons (NFS). Statistical significance determined via Student’s t-test * p ≤ 0.05, ** p ≤ 0.01, *** p ≤ 0.001.

**
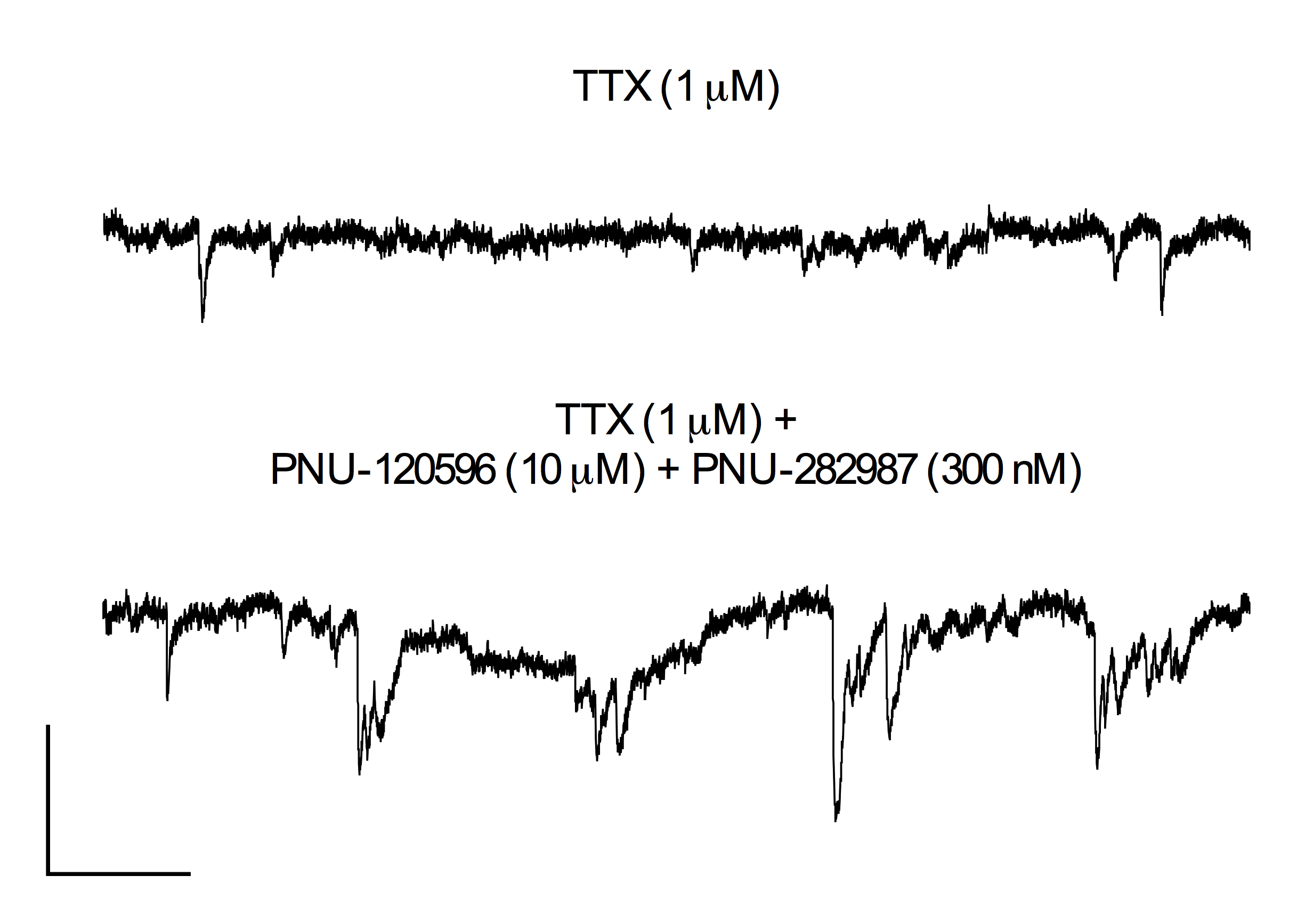
**

**Figure S3. Co-application of PNU-120596 & PNU-228987 in the presence of TTX leads to current fluctuations in Layer V pyramidal neurons.**

Measurement of mEPSCs in the presence of TTX were recorded from layer V pyramidal neurons. Upon co-application of the α7 nAChR PAM (PNU-120596) (10 μM) and agonist (PNU-282987) (300 nM), a large increase in membrane current were observed, coinciding with bursts of mEPSC activity. Instability in the baseline current prevented accurate mEPSC measurement and so a reduced submaximal concentration of PNU-28987 were used for subsequent mEPSC experiments. Scale bar: 25 pA, 250 ms.


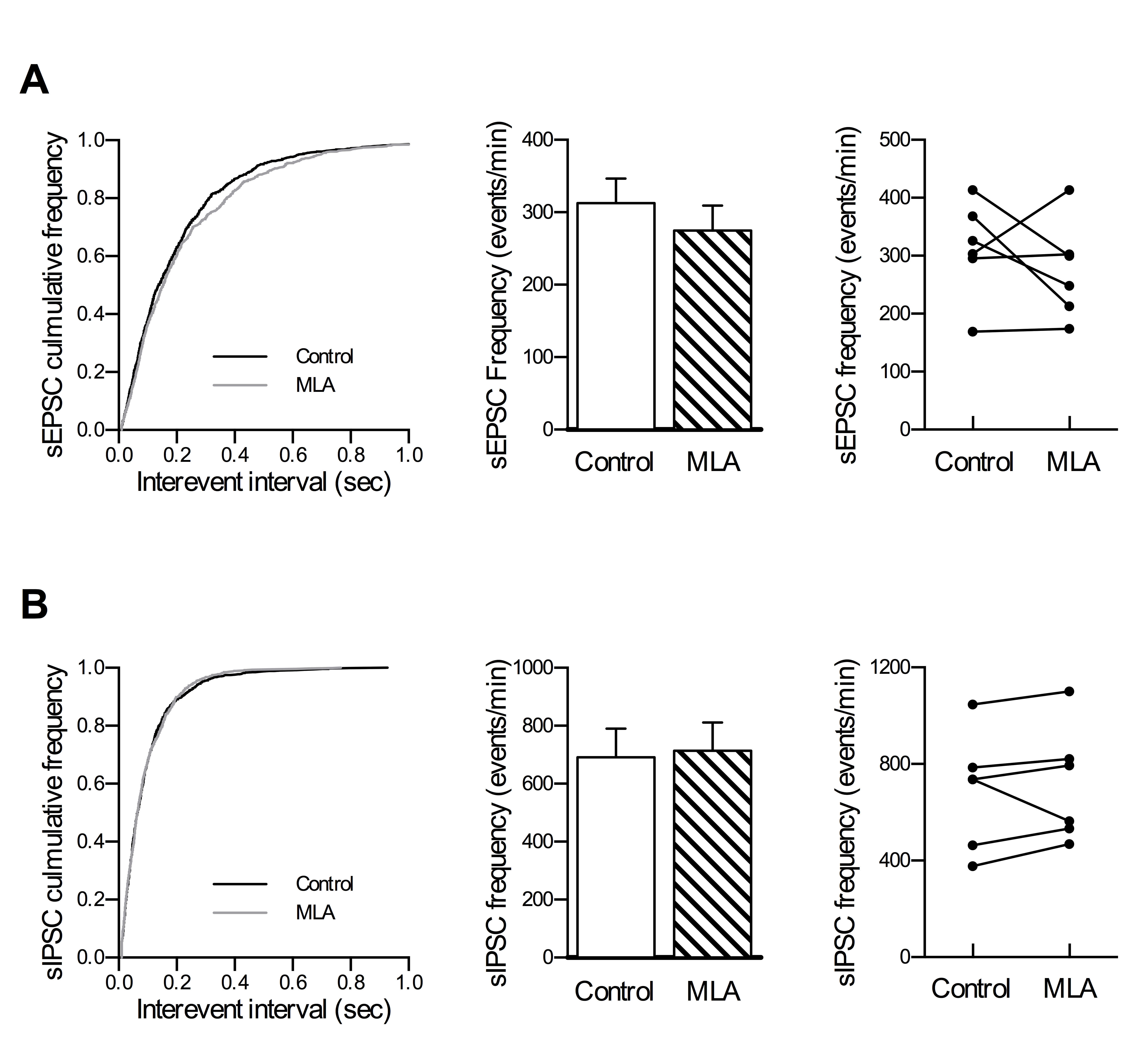


**Figure S4. Effect of MLA application alone on sEPSCs or IPSCs**

Spontaneous excitatory (sEPSC) and inhibitory (sIPSC) post synaptic current frequency was measured in layer V pyramidal neurons in the presence and absence of α7 nAChR antagonist MLA. Cumulative distribution, summary histogram and individual cell frequencies to application of 100 nM MLA for sEPSCs (**A**) and sIPSCs (**B**) (n = 6). For sEPSCs, *p* = 0.04, K-S test. For sIPSCs, p = 0.85, K-S test.


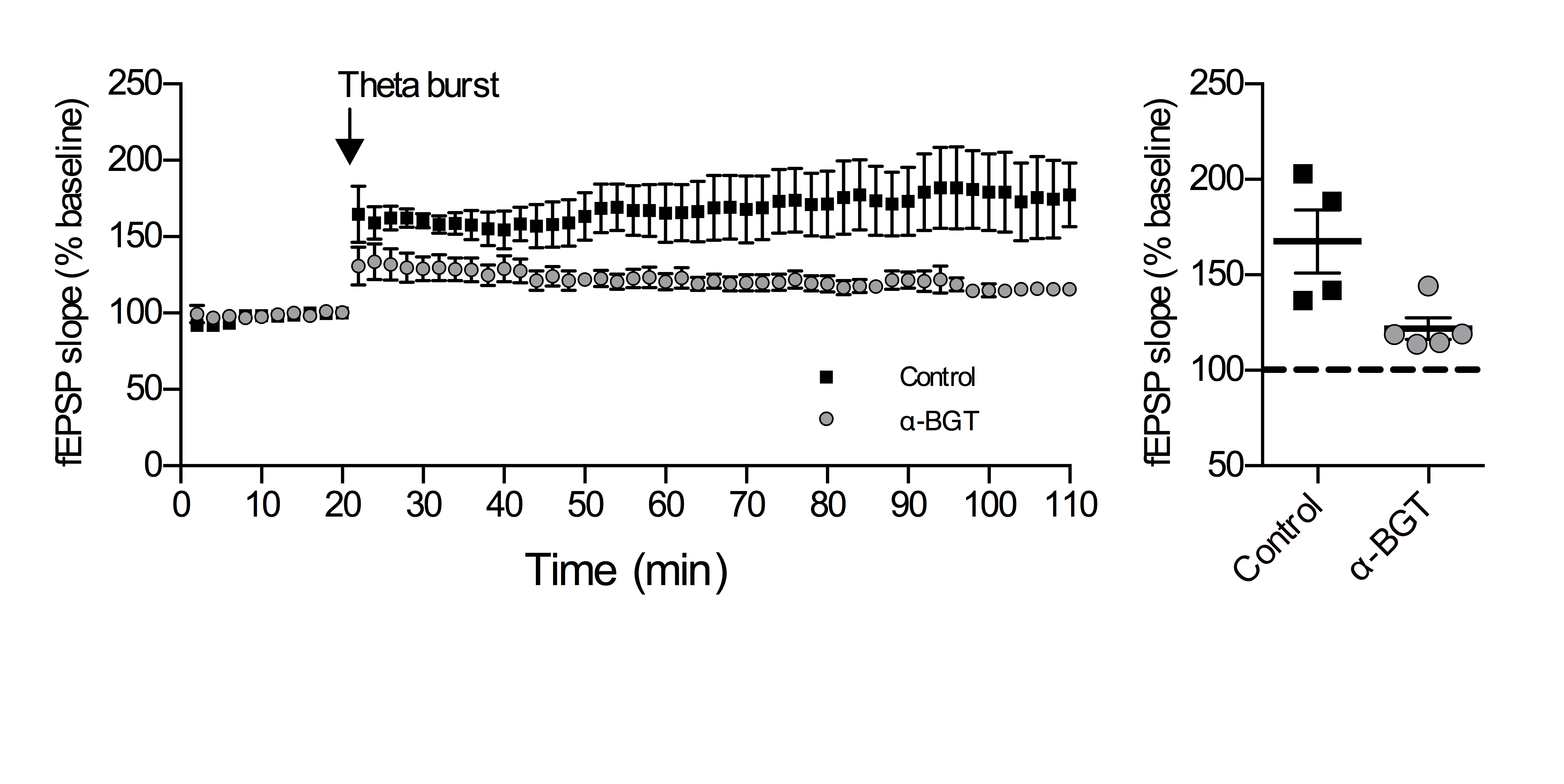


**Figure S5. α7 nAChR antagonism with the irreversible antagonist α-bungarotoxin inhibited the level of theta burst induced long term potentiation (LTP)**

Field EPSPs (fEPSPs) were recorded from prelimbic layer V; long-term potentiation was induced via a theta burst stimulation in layer II/III. Recordings were made in slices incubated for >1hr in the absence (control n = 4) or presence of α-bungarotoxin (300 nM; n = 5) α-bungarotoxin incubated slices had a significantly lower level of LTP from control, *p* ≤ 0.05, Student’s t-test. Histogram shows fEPSP slope 60 mins post-theta burst.
